# Large-scale culturing of the subpolar foraminifera *Globigerina bulloides* reveals tolerance to a large range of environmental parameters associated to different life-strategies and an extended lifespan

**DOI:** 10.1093/plankt/fbae029

**Published:** 2024-06-07

**Authors:** Freya E Sykes, Julie Meilland, Adele Westgård, Thomas B Chalk, Melissa Chierici, Gavin L Foster, Mohamed M Ezat

**Affiliations:** iC3: Centre for ice, Cryosphere, Carbon and Climate, Department of Geosciences, UiT—The Arctic University of Norway, Dramsvegen 201, 9014 Tromsø, Norway; MARUM—Center for Marine Environmental Sciences, University of Bremen, Leoberner Str. 8, Bremen 28359, Germany; iC3: Centre for ice, Cryosphere, Carbon and Climate, Department of Geosciences, UiT—The Arctic University of Norway, Dramsvegen 201, 9014 Tromsø, Norway; Aix Marseille Université, CNRS, IRD, INRAE, CERGE, Technopole Environnement Arbois-Méditerranée BP 80 13545 Aix-en-Provence, Cedex 04, France; Institute of Marine Research, Oceanography and climate research group, Fram Centre, Hjalmar Johansens gate 14, 9007 Tromsø, Norway; School of Ocean and Earth Science, National Oceanography Centre Southampton, University of Southampton, European Way, Southampton SO14 3ZH, UK; iC3: Centre for ice, Cryosphere, Carbon and Climate, Department of Geosciences, UiT—The Arctic University of Norway, Dramsvegen 201, 9014 Tromsø, Norway

**Keywords:** planktic foraminifera, climate change, subpolar, marine calcifier, culture experiment

## Abstract

The subtropical to subpolar planktic foraminifera *Globigerina bulloides* is a calcifying marine protist, and one of the dominant foraminiferal species of the Nordic Seas. Previously, the relative abundance and shell geochemistry of fossil *G. bulloides* have been studied for palaeoceanographic reconstructions. There is however a lack of biological observations on the species and a poor understanding of its ecological tolerances, especially for high latitude genotypes. Here, we present observations from the first extensive culturing of *G. bulloides* under subpolar conditions, including the first low temperature (6–13°C) and variable salinity (30–38) experiments. Carbonate chemistry (pH and [CO_3_^2−^]) was also manipulated. Experimental conditions were chosen to reflect a range of plausible past and future scenarios for the Nordic Seas. We found *G. bulloides* to be tolerant of environmental conditions well outside their optimal range (<10°C, salinity <33, pH <8). Observed life span was up to three months, which was attributed to a microalgal diet. Two alternative life strategies were employed, whereby individuals either experienced rapid growth and death, or a prolonged lifespan with minimal growth and death via slow decay. We posit this could help explain differences in geochemical signals recorded from different size fractions of fossil specimens used for palaeoceanographic reconstructions.

## INTRODUCTION

Planktic foraminifera are calcifying marine protists living in the upper water column of the world’s oceans. They are collectively responsible for 0.1%—3.8% of the global carbonate export flux over the upper 200 m (Knecht et al., 2023), with local and seasonal fluxes up to several orders of magnitude higher (Anglada-Ortiz et al., 2021; Tell et al., 2022). Schiebel (2002) estimated that their contribution to the global open marine flux at 100 m depth could be as high as 56%. The export and post mortem dissolution of their calcium carbonate (CaCO_3_) shell contributes to the carbon and alkalinity pumps (Sulpis et al., 2021), making them valuable contributors to the global carbon cycle. As an individual foraminifer grows, cations substitute into the chemical lattice of its CaCO_3_ shell in a ratio that reflects a range of seawater properties and composition at the time of growth (Nürnberg et al., 1996; Yu et al., 2007; Wit et al., 2013; Allen et al., 2016; Livsey et al., 2020). The sensitivity of the shell chemistry to environmental changes is widely utilized in palaeoceanographic reconstructions by analyzing fossil shells that have settled on the seafloor and been recovered as sediment cores (e.g. Mashiotta et al., 1999; Elderfield and Ganssen, 2000; Ezat et al., 2016). In northern hemispheric subpolar to polar regions, the foraminiferal assemblage is predominantly restricted to *Globigerinita glutinata, Globigerinita uvula, Neogloboquadrina incompta, Neogloboquadrina pachyderma, Turborotalita quinqueloba,* and *Globigerina bulloides* (Husum and Hald, 2012; Schiebel et al., 2017; Anglada-Ortiz et al., 2021), with only the latter four present in significant abundances in waters colder than 9°C (Kucera et al., 2005). As a temperate species that tolerates subtropical to subpolar conditions, *G. bulloides* is of particular interest due to its encroachment into previously “cold water only” territories (Greco et al., 2022).

The Atlantic Ocean—Nordic Seas—Arctic Ocean interface is currently garnering increased scrutiny in the context of contemporary climate change (Glessmer et al., 2014; Rudels et al., 2015; Polyakov et al., 2017; Walczowski et al., 2017; Asbjørnsen et al., 2020; Saes et al., 2022). “Atlantification” of the Nordic Seas and Arctic Ocean is causing warming and salinification, due to changes in the transport of Atlantic Water poleward, with consequent impacts on thermohaline circulation, sea ice formation, heat flux, surface water currents, and ecology (Asbjørnsen et al., 2020; Ingvaldsen et al., 2021; Gerland et al., 2023). Rising temperatures has caused a shift in planktic foraminifera assemblages whereby warm-water species are moving in (Jonkers et al., 2019), resulting in traditionally temperate species being found as far north as 82°N (Anglada-Ortiz et al., 2021, 2023; Greco et al., 2022). Of these, *G. bulloides* is closely linked to Atlantic water and its advection northwards (Volkmann, 2000; Greco et al., 2022), and can be expected to increase in prominence through habitat tracking (Greco et al., 2022). Concurrently, the Norwegian Sea has also seen episodes of local decoupling of temperature and salinity, with simultaneous warming and freshening in the upper 500 m (Mork et al., 2019). It is predicted that Arctic sea ice decline could cause Nordic Seas surface freshening of up to—0.46 psu (Li and Fedorov, 2021), which may temporarily counteract some of the impacts of Atlantification. Additionally, Nordic Seas acidification due to ocean uptake of anthropogenic CO_2_ (Fransner et al., 2022) has the potential to severely disrupt marine life, in particular the marine calcifiers, by hindering growth and biological processes (Kuroyanagi et al., 2013; Davis et al., 2017; Dong et al., 2022).

Despite being cultured in laboratories since the 1970s (see Table I), previous culturing work on *G. bulloides* has predominantly focused on its application as a geochemical proxy (Mashiotta et al., 1997; Lea et al., 1999; Russell et al., 2004; Hönisch et al., 2011; Allen et al., 2016). Biological, metabolic and behavioural observations are scarce, despite these processes contributing to geochemical “vital effects” (Kearns et al., 2023). Furthermore, previous culturing studies were conducted in the subtropics, predominantly coastal California (Spero and Lea, 1996; Hönisch et al., 2011; Davis et al., 2017), and therefore do not replicate the full range of the ecological and biological environments of temperate to subpolar *G. bulloides* specimens. Current published work is based on temperatures ranging from 14.5°C up to 25°C (Adshead, 1967; Russell et al., 2004), and most likely relies on a locally specific genotype that has not been identified outside of offshore California, limiting their global applicability (Bird et al., 2017). The existence of cryptic genotypes with different ecological preferences (Stewart et al., 2001; Morard et al., 2013; Sadekov et al., 2016) therefore represents a significant uncertainty when extrapolating these earlier *G. bulloides* studies to the subpolar North Atlantic Ocean and the Nordic Seas. Even when genotypes coexist, they may exhibit significantly different geochemical signatures despite growing under the same environmental conditions (Sadekov et al., 2016). Furthermore there is an apparent isotopic offset in δ^18^O between *G. bulloides* shells and predicted seawater values (Spero and Lea, 1996) that increases in cooler waters (Daëron and Gray, 2023) and indicates the presence of hitherto under explored biomineralisation processes at low temperatures.

**Table I:** Overview of previous culturing studies on Globigerina bulloides. Ocean basins are simplified from Longhurst et al. (1995) and Tréguer et al. (2021). Subtropical North Pacific East (SNPE), Subtropical North Pacific West (SNPW), Caribbean (CARB), Subpolar North Atlantic (SpNA)

Publication	Location (oceanic basin)	Number specimens	Longevity	Feeding	Size change	Experimental set-up
This study	Norwegian Sea (SpNA)	252 for culture and a further 36 for feeding experiments.	Up to 87 days. Most experiments were ended at day 34.	*Nannochloropsis* (microalgae) solution.	Caught using a 64 μm net. Minimum observed size before growth was 128 μm. Maximum final size 534.53 μm. Mean final size of 312.61 (SD = 69.40) μm	Temperatures 6–9.5°C, salinity 30.4–37.8, pH 7.7–8.35, [CO_3_^2−^] 77.1 and 224.5.
(Adshead, 1967)	Coast of s. California (SNPE)	Unclear, collected over the course of six one-day cruises.	The largest specimens generally died after the first week. “*Young*”, transparent specimens were usually the most successful and survived up to 3 months.	Unknown mixture of planktic algae, collected during the cruises and then furthered cultured in the laboratory to keep it fresh. Found to be tolerant of variations in algal density.	Most caught using a 253 μm net. Some may have been picked from a 64 μm mesh. No final sizes given.	Temperatures of 14.5–16°C.
(Spero and Lea, 1996)	Santa Barbara Basin and San Pedro Basin, CA (SNPE)	166	Considered dead when released gametes and dropped spines.	1-day-old *Artemia* sp. nauplius daily. Two strains of *Artemia* were used.	Dive-picked “*small*” specimens. Average initial size was 243 ± 52 to 313 ± 59 μm. Average final size was 430 ± 39 to 518 ± 39 μm. Greatest increase at temperature 22°C Chamber addition occurred over 4 to 5 days.	Temperatures of 16 and 22°C.
(Mashiotta et al., 1997)	San Pedro Basin, CA (SNPE)	124 were used for analysis, 16 of these were then rejected due to suspected sample contamination.	Considered dead after gametogenesis. Culture period of 7–10 days	Fed a 1-day-old *Artemia* sp. nauplius every other day.	Dive-picked “*small*” specimens. Two to four new chambers. After culturing weighed between 7 and 10 μg.	22 °C water bath, 12:12 hour light:dark cycle.
(Uhle et al., 1997)	Coast of Santa Catalina Island. CA (SNPE)	Unspecified	5–10 days. Individuals were removed from culture just prior to gametogenesis.	Fed a single *Artemia* sp. nauplius daily.	Collected “*juvenile”* specimens by diving. All individuals at least doubled in size.	Maintained at an ambient seawater temperature of 20 ± 1.5°C
(Bemis et al., 1998)	San Pedro Basin, CA (SNPE)	Approximately 230	Cultured until gametogenesis	Fed a single *Artemia* sp. nauplius every other day.	Handpicked by divers from the surface 2–6 m	Maintained at temperatures of 15°C, 17, 19, 22, and 24 ± 0.2°C. Illumination was on a 12:12 hour light:dark cycle
(Lea et al., 1999)	San Pedro Basin and Santa Barbara Channel, CA (SNPE)	19 analyses, unclear how many specimens used.	Considered dead after gametogenesis. 7–10 day culture period.	Fed a 1-day-old *Artemia* sp. nauplius every other day.	Handpicked “*small*” specimens. 2–4 new chambers.	Temperatures 16, 22, and 25°C, pH 7.6 and 8.5. 12 hour light: 12 hour dark cycle.
(Russell et al., 2004)	San Pedro Basin, CA (SNPE)	Unclear	Experiments ended after gametogenesis.	Fed a single 1-day-old *Artemia salina* nauplius every other day.	Handpicked “*small*” specimens. Grew 1–four new chambers.	Variable [CO_3_^2−^] of 76–468 μmol/kg, temp 25°C.
(Hönisch et al., 2011)	San Pedro Basin, CA (SNPE)	35 chambers were analyzed, unclear how many individual specimens.	Died after gametogenesis. 7–10 day culture period.	Fed a 1-day-old *Artemia* sp. nauplius every other or 3rd day.	Hand collected “*juvenile*” specimens. Two to four new chambers.	pH 7.61–8.52.
(Kuroyanagi et al., 2013)	San Pedro Basin, CA (SNPE)	49	Classified as undergoing gametogenesis when shed spines (average time of 4.7–6.8 days). < 40% died without gametogenesis.	Fed a 1-day-old *Artemia* sp. nauplius every day.	218.5 1SD 37.1–283 1SD 30.9 μm mean initial length. Mean final length of 354.6 1SD 50.2–436.1 1σ 60.1 μm. Final mean shell weight of between 3.4 and 7.7 μg.	Experiments on dissolved oxygen concentration.
(Allen et al., 2016)	Santa Catalina Island (SNPE), Isla Magueyes (CARB)	Around 30 individuals per treatment, so ~90 in total.	1–3 weeks	*A. salina* every other day.	Hand collected “*juvenile*” specimens. Average calcification rate of 0.2–0.4 μg/day.	Temperatures of 18.5 and 22.5°C. Total pH between 8 and 8.3.
(Davis et al., 2017)	Bodega Head, CA (SNPE)	At least 10–12 per treatment.	Up to 12 days. Only 30% of pH 7.5 specimens regrew spines after the shock of collection.	Fed 1-day old freeze-killed *Artemia* sp. nauplius every other day.	Caught with 155 μm net. Longest shell dimension of around 200–300 μm per depending on treatment. Decreased calcification at low pHs (<7.7).	pH 7.5–8.3, 16°C.
(Hori et al., 2018)	Sagami Bay, Japan (SNPW)	6	Death defined as loss of spines and lack of pseudopod activity. Survived 10–20 days.	Fed *Artemia* sp. nauplii.	Caught using plankton net of undefined mesh size. One individual grew one chamber and three individuals grew two chambers in culture.	Temperatures of 19, 21, 23, and 25°C.
(Kuroyanagi et al., 2019)	Sagami Bay, Japan (SNPW)	7	4 days.	NA	Caught using 63 μm plankton net. Initially between 228 and 345 μm.	Euxinic condition experiments. 19.5°C.

The potential impacts of ocean acidification and salinity anomalies on *G. bulloides* remain uncertain, as little research has been conducted on the former (Davis et al., 2017), and none on the latter (see Table I). This lack of investigation poses challenges in discerning the biological consequences of these environmental changes for *G. bulloides*. Additionally, the dietary requirements of *G. bulloides* are a topic of ongoing discussion. While commonly fed *Artemia* spp. nauplii (larval stage) in culture, it is suggested that in the open ocean*, G. bulloides* may exhibit herbivorous tendencies, particularly in colder waters (Grigoratou et al., 2021). Tracer experiments have also hinted at a preference for microalgae when multiple food sources are available (Lee et al., 1966). The implication of different feeding regimes on cultured specimens has not been investigated despite it likely having a large role to play in a specimen’s adaptation to life in culture and subsequent tolerance to different environmental parameters. Recent findings of previously undescribed behaviors, such as ectoplasmic structures (Greco et al., 2023) further underscore the necessity for comprehensive reportage of *G. bulloides* behaviour in culture.

This study seeks to bridge the gap between the biological activity of *G. bulloides* and earlier laboratory-based studies for the development of geochemical proxies. This is accomplished by presenting comprehensive observations from an extensive set of culturing experiments on *G. bulloides* specimens collected from the subpolar ocean, including the first experiments on its tolerance to salinity variability and low temperatures (below 14.5°C). Additionally, we investigate the biological response and tolerances to alterations in carbonate chemistry, in particular pH and carbonate ion concentration ([CO_3_^2−^]). We also discuss the implications of diet and observed mortality and growth rates and as such build a foundation for a holistic understanding of the species’ responses to varying environmental parameters, paving the way for enhanced future investigations in both biological and geochemical disciplines.

### Review of previous *G. bulloides* culturing

A review of previous culturing work carried out on *G. bulloides* is presented in Table I. Features are highlighted that are especially relevant to this study including sampling method, mortality, feeding, and experimental conditions. The sampling locations are defined based on ocean basins simplified from Longhurst et al. and Tréguer et al. (2021). All but four of the previous studies used specimens from the Subtropical North Pacific East and only one included Atlantic individuals (Allen et al., 2016). Temperatures cover a range from 6 to 35°C, salinities from 30.4 to 37.8, and pH 7.5 to 8.52. Where specified, the number of specimens used in each culturing study varied from 6 to 288. Culturing generally ended by week three, but in two cases some specimens survived for up to 3 months.

## METHODS AND MATERIALS

### Foraminifera and water collection

Sampling was carried out in late June 2022 onboard RV Helmer Hanssen in the Norwegian Sea (Ezat et al., 2022). Physical variables (salinity and temperature) were measured in seawater collected from 68^o^14’N 10^o^12’E at 150 m water depth using a Conductivity-Temperature-Depth CTD (Seabird-911 Plus) Rosette system equipped with twelve 10-L Niskin bottles. Seawater from the Niskin bottles was immediately filtered through a 2 μm nitrate cellulose filter. Water salinity and pH were obtained by the CTD and Metrohm 914 pH-meter with an Aquatrode Plus with integrated Pt1000 temperature sensor pH-electrode, respectively. These values were used as the basis for experimental water manipulation (see *Experimental water manipulation, monitoring, and analyses*). Plankton sampling was carried out from the upper 75 m at 66°58’N 7°38′E using a WP2 vertical plankton net (HydroBios) with 64 μm mesh size. In total, 288 specimens were placed into culture. Healthy juvenile (small with a low number of chambers) specimens were picked using brushes and placed into individual 75 mL Falcon flasks with the appropriate experimental water (see Table II). The health of the individual was assessed by the presence of rhizopods, spines, and coloured (green/brown) cytoplasm. Ambient conditions at 25 m depth at the collection site were 8.7°C, salinity 35.3, and pH total scale (pH_T_) 8.10. The full range of experimental conditions are detailed in Table II (final values for water chemistry come from analyses done at the Institute of Marine Research in Tromsø, Norway, see *Experimental water manipulation, monitoring, and analyses*). Light conditions in culture were set to a 24-hour cycle split into 3-hour intervals of varying light intensities (0, 15, 26, 37, 45, 37, 26, 15 μmol m^−2^ s^−1^, using white LED bands), which mimicked the daily rhythm at the collection site.

**Table II:** Overview of the different water parameters for each experimental treatment and number of individuals (n) in each treatment. ID refers to their in text identifier, T is temperature, pH_T_ is pH total scale, DIC is dissolved inorganic carbon, A_T_ is total alkalinity, Ωca is calcite saturation, and n is number of specimens in that treatment

ID	T (°C)	pH_T_	Salinity	DIC (μmol/kg)	[CO_3_^2−^] (μmol/kg)	A_T_ (μmol/kg)	Ωca	(n)
T6	6	8.0	35.4	2244	110.2	2373	2.6	25
T13	13	7.9	35.4	2240	113.8	2376	2.73	27
S30.4	9.5	7.9	30.4	1942	93.1	2054	2.3	23
S32.5	9.5	8.0	32.5	2043	120.1	2197	3.0	21
S37.8	9.5	8.1	37.8	2324	185.0	2572	4.4	20
pH 7.7	9.5	7.7	35.4	2173	71.0	2233	1.7	25
pH 7.8	9.5	7.8	35.3	2176	84.7	2263	2.0	18
pH 8.1	9.5	8.1	35.3	2180	149.8	2378	3.6	33
pH 8.3	9.5	8.3	35.4	2268	229.1	2581	4.7	21
CI77	9.5	7.9	36.7	1614	77.1	1718	1.8	20
CI225	9.5	8.0	35.2	3378	224.5	3636	5.5	19

The different experimental treatments will henceforth be referred to by their abbreviated identifier (ID). In text individual specimens may be identified as ID_n where ID is the experimental treatment and n is the individual specimen number within that treatment. The full details of all the water parameters associated with each experiment and its ID are described in Table II. Specimens in treatments pH 7.7 and S30.4 were kept in acclimatisation water for 24 hours (pH 7.8/[CO_3_^2−^] 84.7, and salinity 32.5 respectively) before being placed into the final culturing conditions, to minimize shock to the organisms. Due to equipment limitations onboard RV Helmer Hanssen, treatment T13 was kept at 9.5°C until arrival at the laboratory. In Tromsø all specimens were transferred to incubators (Friocell 222 EVO incubators with LED light shelves, with a temperature precision of ± <0.5°C and<0.2°C in space and time, respectively) at the Department of Geoscience, UiT—The Arctic University of Norway.

### Experimental water manipulation, monitoring, and analyses

Salinity and carbonate chemistry were manipulated so that salinity, pH and/or [CO_3_^2−^] represented the range of oceanographic conditions of interest (Table II). For salinity, filtered seawater was partially frozen at −20°C, and the ice removed. The resultant brine was used to increase salinity, and the melted ice to decrease it. Acid or base addition was used to adjust pH. Hydrochloric acid (HCl) was added to decrease pH, and sodium hydroxide (NaOH) to increase it. This alters total alkalinity (A_T_) while having minimal effect on the total dissolved inorganic carbon (DIC). For decoupled carbonate chemistry, [CO_3_^2−^] was altered without changing pH. For lower [CO_3_^2−^], artificial seawater (distilled water and salt) was added. For high [CO_3_^2−^], NaHCO_3_ was added.

Experimental water was stored in black 20 L jerry cans at <5°C to minimise any biological activity. Water salinity and pH was monitored throughout the experiments using a handheld AdolfR refractometer (accuracy ±0.20%), and Metrohm 914 pH-meter (accuracy ±0.003 pH), respectively. Samples from each treatment were analysed for water chemistry at the Institute of Marine Research in Tromsø, Norway. Sample water was taken from the different treatments throughout the experiment and upon its termination. The water was stored for analysis in 250 mL glass bottles at 6°C and poisoned with 60 μL HgCl_2_ to prevent further biological activity. Salinity was measured using a WTW Cond 330i conductivity meter, with a precision and accuracy of ±0.05. Experimental water pH_T_ and carbonate ion concentration ([CO_3_^2−^]) were calculated based on pairs of measured total alkalinity (A_T_), and total DIC, salinity, and temperature using the chemical speciation software CO2SYS (Pierrot et al., 2006). The carbonate dissociation constants of Dickson and Millero (1987) were used in CO2SYS on pH_T_. A_T_ was measured using potentiometric titration with 0.1 N hydrochloric acid and DIC was measured using coulometric titration from acidified samples following standard protocols (Dickson et al., 2007). Accuracy and precision were ensured based on replicate measurements of Certified Reference Material (USA) and was ±2 μmol kg^−1^ for both A_T_ and DIC. All values were measured at room temperature, and pH was subsequently corrected to experimental temperature using CO2SYS.

### Culturing observations and feeding

While in culture, specimens were observed at a minimum twice weekly, and observations about size, cytoplasm density, rhizopod extension, spine density, and behaviour recorded. They were regularly imaged using an inverted microscope (Zeiss AxioVert 0.1, equipped with an AxioCam 208 colour camera). An individual was labeled dead when it appeared to be empty of cytoplasm and was no longer buoyant. In previous culturing studies, the complete loss of spines has also been used to indicate death (Spero and Lea, 1996; Kuroyanagi et al., 2013). As this study will later discuss, however, this could not be relied on to occur consistently, with specimens observed to lose spines but continue to live (as defined by the existence of observable rhizopodial activity), or conversely die while still retaining spines. By day 34 all experiments were ended, except for S37.8, pH 8.1, and CI77, which were continued until day 87 (numbers of still living at day 34 = 6, 4, and 2, respectively) to investigate their longevity in culture. Where individuals had not died naturally by the end of the experiment, they were killed by placement in distilled water. After death, each individual was photographed, and its longest axis (maximum linear dimension of the shell whorl, as described by Spero and Lea, 1996) was measured in the Zeiss software ZEN 3.3. Due to time and equipment constraints onboard RV Helmer Hanssen, it was not possible to take initial size measurements. Instead, a sub-sample of 30 specimens were taken from the plankton net and instantly killed before being divided into “small” and “large”. The small specimens were considered the size equivalent to the healthy juvenile specimens that had been placed into culture and were measured once back to Tromsø. It is assumed that these net samples represent an initial size range for the cultured specimens (*n* = 8, mean = 230, ± 43 1SD, range = 163 to 277 μm).

Feeding experiments were carried out onboard with a subset of *G. bulloides* individuals prior to the onset of culturing at UiT, using microalgae (*Nannochloropsis* spp. mix), fresh *Artemia* sp. Nauplii, and frozen *Artemia* sp. Nauplii. It was determined that *G. bulloides* readily accepted microalgae as a food source and thus this was used throughout the experimental period. This significantly increased the number of individuals it was possible to manage in culture, relative to using *Artemia* sp. Nauplii. It may also better reflect their diet in the marine environment they were collected from, as cold water *G. bulloides* are likely primarily herbivorous (Grigoratou et al., 2021). All individuals were fed every second day with 10 μL of a freshly autoclaved solution of microalgae (50 μL *Nannochloropsis* spp. concentrate and 200 mL filtered seawater). Autoclaving killed the *Nannochloropsis* spp., preventing algal blooming in the culture water.

### Statistical analysis

Statistical analyses were carried out using MATLAB R2022b and IBM SPSS Statistics (version 29). Percentage mortality over time in culture was statistically modelled using the logistic equation $=a/(1+\mathit{\exp}\left(-b\ast \left(x-c\right)\right))$, using a nonlinear least squares method to extract coefficient values. The logistic growth model is commonly used in population statistics as it assumes an eventual carrying capacity for the population beyond which values can no longer increase (Tsoularis and Wallace, 2002), making it suited to plotting population mortality over time. The R-squared value was obtained to describe goodness of fit and 95% prediction bounds plotted to limit the expected bounds for mortality on a particular day. Pearson’s correlation ($p$) was calculated to characterise the relationship between final longest axis and longevity in culture. One-way ANOVA and Tukey’s honestly significant difference test was used to check for significant differences in final longest axis length between the treatments, and the relationship between spine loss and longevity in culture.

## RESULTS

### Mortality

**Fig. 1:**
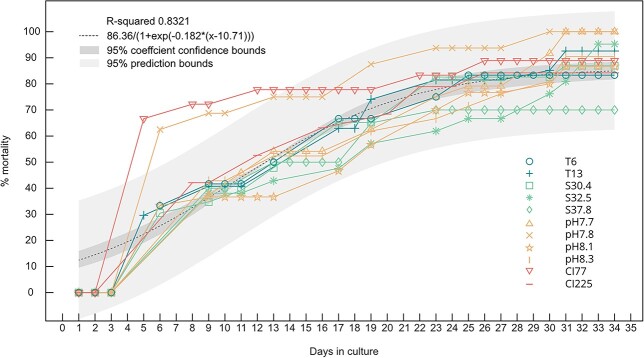
Percentage mortality in each treatment as a function of days in culture. Symbols refer to the different treatments which are detailed in Table I. The dark gray-shaded area indicates the 95% coefficient confidence bounds and the light gray-shaded area the 95% prediction bounds. The best fit logistic expression y = 86.36/(1 + exp(−0.182*(x-10.71)) is marked with dashed line.

The mortality rate was consistent between all treatments, except for pH 7.8 and CI77 which had a large spike in mortality over the first 5 days relative to other treatments (Fig. 1). The trend for all other treatments is described by $y=a/(1+\mathit{\exp}\left(-b\ast \left(x-c\right)\right))$, whereby the coefficients (with 95% confidence bounds) are $a$ = 86.36 (83.53, 89.19), $b$ = 0.182 (0.1606, 0.2035), $c$ = 10.71 (10.03, 11.38), with an R-square value of 0.833. Thus, where $x$ is the number of culture day, we calculate a predicted mortality for a Norwegian Sea juvenile *G. bulloides* for that given day under comparable culturing conditions. By day 34 the mortality within all treatments, S37.8 excepted, had reached at least 80%. Notably, there was a delay of several days before any mortalities occurred, however, as those that did die earliest tended to show signs of decline immediately after picking, it implies it was still shock related in these cases.

Although most experiments were ended at day 34, six specimens were kept alive for a total of 87 days. Had the other experiments not been ended early, it is possible that more individuals could have reached this milestone or surpassed it.

### Mortality modes

**Fig. 2:**
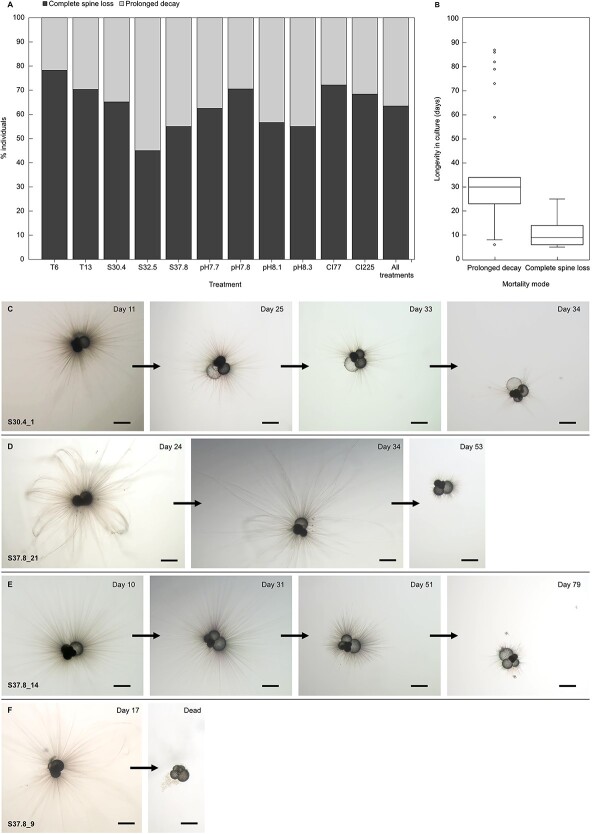
Mortality modes in cultured *Globigerina bulloides*. (A) and (B) present mortality mode as a function of treatment and of longevity in culture. Plates (C), (D), and (E) demonstrate the prolonged decay, whereby cytoplasm recedes from the younger chambers, and rhizopods and spines decrease in density and length over a prolonged period. Even when apparently dead, the individual retains some sparse spines and potentially white cytoplasm in the earliest chambers. (F) shows an individual that was apparently healthy on day 17 but when next observed 2 days later, had lost all cytoplasm and spines. Scale is 200 μm in all images.

There was a distinct split between individuals that died with complete spine and cytoplasm loss (63%), and those which deteriorated slowly over a prolonged period, often retaining sparse and/or stubby spines even when no longer maintaining cytoplasm or rhizopods (37%). Individuals dying earlier tended to rapidly lose all spines and cytoplasm, whereas those surviving longer retained sparse or shortened spines, and in several cases never fully lost them, despite an absence of cytoplasm or rhizopodial activity (Fig. 2). The greatest percentage of complete spine loss was seen in T6 (78%), and the lowest in S32.5 (45%). One way ANOVA testing on the relationship of longevity in culture to spine loss, gave an F value of 175.506 and *p* value of < 0.001, implying lifespan is strongly correlated with the occurrence of complete spine loss at death. Death with complete spine loss occurred over a much more rapid time scale, and individuals could be apparently healthy on one day and dead the next, whereas the prolonged decay may be observed over several weeks.

### Growth

Growth could be observed by an increase in shell size, the addition of thinner or transparent final chambers, and/or misshapen final chambers, the latter being a recurrent artifact of culturing (Westgård et al., 2023). The largest specimen had a final longest axis of 535 μm and the mean length across all treatments was 313 (± 69 1SD) μm. Final size by treatment is listed in Table III. It appeared that any chamber addition occurred within the first 9 days of culture. This does not discount the occurrence of further calcification however and shell thickening may have continued without chamber addition.

**Table III:** Size range in final longest axis (μm) for specimens grown in culture

ID	Min	Max	Median	Mean
T6	195	456	320	319
T13	225	491	305	318
S30.4	194	398	299	299
S32.5	214	410	309	306
S37.8	185	475	289	296
pH 7.7	171	439	301	307
pH 7.8	192	424	323	318
pH 8.1	165	403	292	290
pH 8.3	217	535	328	341
CI77	216	485	345	342
CI225	198	474	300	322

**Fig. 3:**
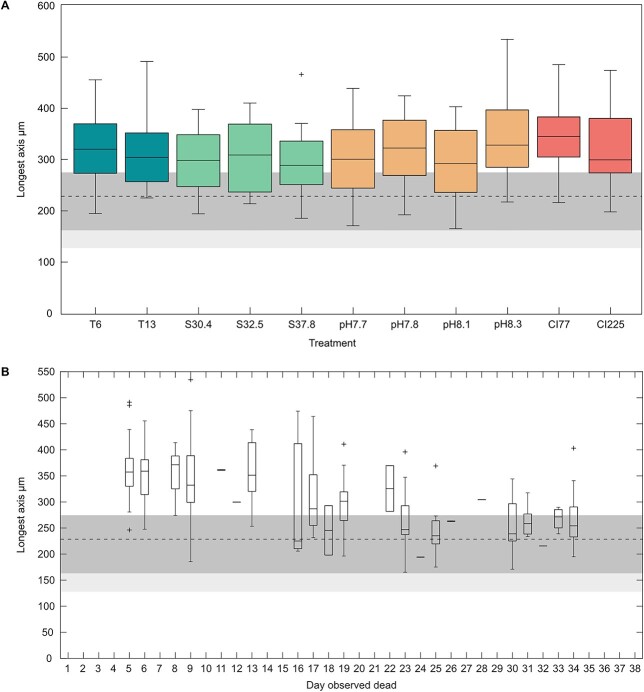
Final longest axis length per treatment (A), and final longest axis length as a function of the day the specimen was observed dead (B). The dark gray shading indicates a theoretical initial size range based on plankton net sub-sampling. The mean of these values is marked as the dashed line. The light gray shading extends this range for initial sizes based on the minimum measured longest axis during the culturing period. Boxplots show median, the lower and upper quartile range, and minimum/maximum values. Outliers (greater than 1.5 times the interquartile range) are marked as +.

ANOVA testing found no statistically significant difference in the length of the longest axis between any of the treatments (*F* = 1.312, *p*-value 0.22). There was, however, a negative relationship between longest axis and mortality, whereby the specimens with the greatest final longest axis died significantly earlier than those with a shorter final longest axis (Fig. 3). When Pearson’s correlation was applied this gave a $r$ value of −0.55 that was significant at the <0.001 level. It seems the specimens can be roughly split into two groups, whereby the longest axes are found on individuals that died before day 18 and the shortest on individuals dying afterwards.

### Feeding

**Fig. 4:**
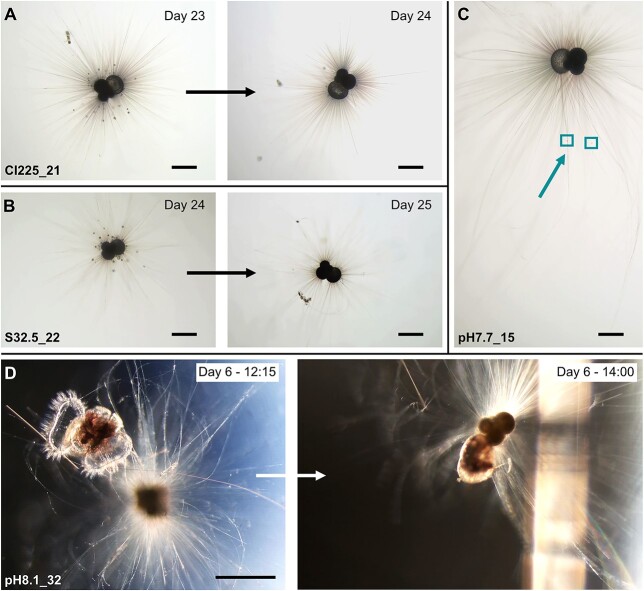
Feeding documented in four different specimens. (A) and (B) show concentrated microalgal particles around the main foraminiferal body. The following day the particles were gone, presumably consumed. (C) displays extended rhizopods to increase surface area and increase food encounters. Microalgae particles trapped in the rhizopods are highlighted. (D) shows the specimen catching a live pteropod. At 12:15 the pteropod was still alive and struggling, but 2 hours later was apparently dead and being consumed, rhizopods having been retracted to bring it closer to the foraminiferal body. Scale is 200 μm in all images

Specimens were documented feeding on the microalgae *Nannochloropsis* while in culture. Food uptake was observed to be associated with extensive rhizopodial activity (Fig. 4). In addition, opportunistic carnivory was also displayed in a foraminifer from treatment pH 8.1 (Fig. 4. D). In this case the specimen (pH 8.1_32) had been transferred to its culture bottle with a live pteropod. When first observed the pteropod was still alive and struggling, entrapped within the foraminiferal rhizopods. Within 2 hours, however, the rhizopods had been retracted, and the pteropod presumably died (inactive) and was being consumed. In another case, apparent cannibalism was observed between two specimens. Overall there was no decrease in longevity relative to earlier studies (Hönisch et al., 2011; Allen et al., 2016; Hori et al., 2018), which we take as evidence that feeding on microalgae as opposed to *Artemia* spp. had no effect on the viability of culture *G. bulloides*.

### Spine regrowth and maintenance

Spine regrowth and maintenance in the first few days after picking can be used as an indicator of recovery after stress during capture (Davis et al., 2017). Initial rates of spine breakage may have been elevated due to using brushes during the initial collection. The recovery and maintenance of spines is described in Table IV and was based on the number of individuals with spines on the third day in culture. It was lowest in treatments CI77 (35%) and pH 7.8 (44.4%). For the other treatments regrowth and/or maintenance was around 65%, with S30.4 and pH 7.7 having values of 100 and 84%, respectively.

**Table IV:** % spine regrowth and/or maintenance on the third day in culture by treatment

Treatment	% spine regrowth and/or maintenance
T6	64
T13	66.7
S30.4	100
S32.5	66.7
S37.8	65
pH 7.7	84
pH 7.8	44.4
pH 8.1	66.7
pH 8.3	62
CI77	35
CI225	73.7

### Ectoplasmic structures

**Fig. 5:**
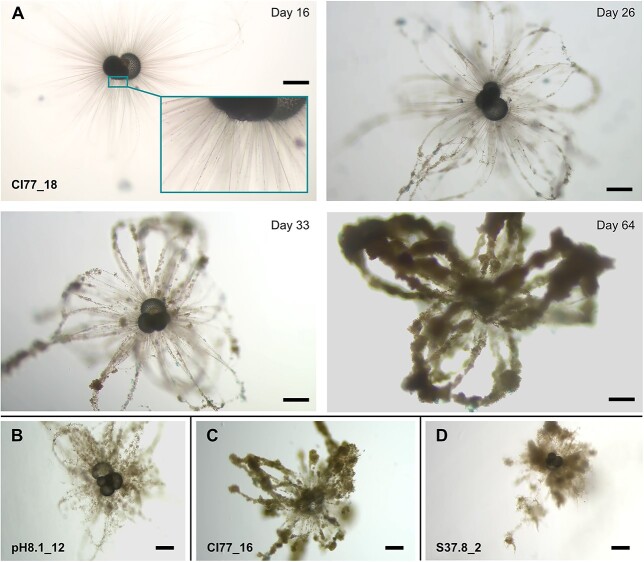
Formation of twig-like ectoplasmic structures. (A) shows their development over time in CI77_18. Their onset was first noted on day 16 with small particles being retained on the spines/rhizopods (seen in inset). Over the following months they developed into twig-like permanent structures This specimen is also shown in Greco et al. (2023), labeled GcLow_18. (B), (C), and (D) show the formation of similar structures at different levels of development in other individuals. Scale bar is 200 μm in all images.

Ectoplasmic structures, or twigs as described by Greco et al. (2023), were first noted as the aggregation of food particles along the rhizopods and spines of specimen CI77_18 on day 16 (Fig. 5). Over the 3-month culturing period this aggregation developed into large, looping structures that eventually enveloped the entire organism, using anatomising spines as an initial skeleton. It appeared that once a particle was in place, it was retained within the structure rather than consumed by the specimen. The structures were so extensive as to be visible without the aid of a microscope. The specimen continued to be suspended in the culturing flask during their development, suggesting they had no negative effect on buoyancy. After 25 and 50 days, such structures also formed to a lesser degree in S37.8_2 and CI77_16, respectively. It is possible that further individuals could have formed ectoplasmic structures had they also been cultured beyond a month, as they were mostly formed after day 34. As an effort was made to keep all treatment water clean of other biological material, it seems unlikely that specimens were able to construct such extensive structures just by chance encounters with contaminants in suspension. That other specimens in the same treatment waters did not develop ectoplasmic structures would also indicate that for those which did, it was an active rather than passive process.

## DISCUSSION

### Mortality and adaptability

Specimens demonstrated unexpected longevity when compared to earlier studies. Previous culturing studies reported that *G. bulloides* did not survive in culture beyond 2 to 3 weeks, with less than 10 days not being uncommon (Mashiotta et al., 1997; Hönisch et al., 2011; Kuroyanagi et al., 2013; Hori et al., 2018). The one exception was Adshead (1967), who also sustained individuals for up to 3 months. Earlier works handpicked culture specimens by diving (see Table I). The advantage of diving is that it is less damaging of the delicate foraminiferal calcite, spines, and cytoplasmic structures, thereby decreasing stress and the recovery time needed after capture (Bé et al., 1977). However, it may also have created a bias towards larger, and therefore older, individuals that consequentially die sooner in culture. Where reported, the average initial size of “dive” captured *G. bulloides* was between 218.5 and 313 μm (Spero and Lea, 1996; Kuroyanagi et al., 2013). Based on plankton net sub-sampling and observation our initial sizes varied between 128 and 277 μm. Other net-based studies of *G. bulloides* (Adshead, 1967; Davis et al., 2017; Hori et al., 2018) had survival rates that were equal to or better than diving; however, a targeted comparison of different planktic foraminifera species also found very little difference in overall mortality between diving and net tows after the first 5 days (Bé et al., 1977).

A further variable that may be influencing *G. bulloides* mortality is feeding. Standard practice in studies on *G. bulloides* (Spero and Lea, 1996; Lea et al., 1999; Hönisch et al., 2011; Davis et al., 2017; Hori et al., 2018) has been to feed newly hatched *Artemia* spp. nauplii daily/every second day, which although providing a high energy and nutritional diet (Léger et al., 1987), is also time consuming to deliver to each cultured individual. Feeding in this way typically involves placing the *Artemia* on the spine/rhizopodial network of the foraminifera, or in the near vicinity of it (Bé et al., 1977; Dong et al., 2022) sometimes by manipulating the foraminifera themselves, which exposes them to repeated stress. In contrast, pipette feeding with microalgae into the culturing material was rapid, minimised contact with the culturing specimen and reduced the time they were kept outside of the incubator. Only one other culturing study on *G. bulloides* could be identified where they had also been fed an algae mix (Adshead, 1967), and this was also characterised by an elevated survival period (up to 3 months). Furthermore, it is possible that algae better reflects their diet in the marine environment they were collected from, especially for cold water, high-latitude individuals (Grigoratou et al., 2021). Early work on *G. bulloides* using different radioactively labeled food sources found a preference for microalgae (Lee et al., 1966). Conversely, the presence of varied organisms within the cytoplasm of *G. bulloides* individuals, including bacteria, algae, diatoms, and coccolithophores, indicates a diverse and opportunistic diet (Bird et al., 2017). Beyond food source, survival rates in culture can clearly be impacted by the amount of feeding. Daily feeding on the planktic foraminiferal species *Globorotalia truncatulinoides* with *Artemia* sp. nauplii resulted in premature death, whereas greater time intervals between feeding resulted in an average lifespan of over a month (Anderson et al., 1979). When allowed to feed at its own discretion regular chamber addition every 24–48 hours was observed (Anderson et al., 1979). Daily feeding intervals of *Hastigerina pelagica* resulted in more regular gametogenesis but a shorter mean survival time than six day feeding intervals (Anderson et al., 1979). In *Globigerinoides sacculifer* daily feeding with *Artemia* sp. nauplii increased growth rate but lead to decreased longevity by the earlier onset of gametogenesis (Caron et al., 1982). It appears that the two-day feeding regime used in this study avoided overfeeding while still ensuring a consistent food supply. As *G. bulloides* are seemingly indifferent to whether given a herbivorous, omnivorous, or carnivorous diet, it seems unlikely that the diet itself controls mortality, but rather the frequency/amount and method of feeding, and any resultant stress this causes.

### Elevated mortality in pH 7.8 and CI77

There is no obvious connection between pH 7.8 and CI77 which could explain why the mortality in these treatments follow such a different trajectory to the others. The two treatments were picked on different days, came from different nets, and the treatments picked alongside them showed no elevated mortality. They also have a rate of recovery and maintenance of spines that is around half that of the other treatments, suggesting that they were stressed from the start of the culturing period. For pH 7.8 it seems unlikely that the low pH or [CO_3_^2−^] can be ascribed as responsible, as specimens of pH 7.7 were also placed in the same solution of pH 7.8 water for 24 hours as part of the acclimatisation process, before being placed under even more acidic conditions, without seemingly suffering any ill effect. A previous study investigating the impact of acidification on *G. bulloides* found the ability to regrow spines decreased in parallel with decreasing pH, however, in that study it was the lowest pH that had the lowest recovery (Davis et al., 2017). While the jump in mortality coincides with the date they were moved into the incubator, this is probably not the cause, as deterioration was already recorded from Days 2 and 3.

In the case of CI77, it may be that the shock of being placed in low carbonate water with ~40% distilled water inflated the death rate. Furthermore, distilled water may be lacking in the nutrients, micro-bota and ions present in seawater that foraminifera require (Westgård et al., 2023). When cultured in water with low [CO_3_^2−^] values (50–100 μmol kg^−1^), *G. bulloides* have been reported to have significantly lower oxygen consumption than at higher values (Davis et al., 2017) suggesting inhibition of critical metabolic processes which could explain the initial high mortality. Why a similar phenomenon is not seen in pH 7.7, which had equally low carbonate value is less clear. It may be that the stepwise acclimatization for these specimens decreased the shock factor. It cannot be ruled out that the increased mortality was due to the initial selection of foraminifera from a particular net trawl.

### Longevity-shell size relationship

Despite evidence for growth, and final longest axis lengths of up to 535 μm, most chamber addition appeared to occur within the first week. This is not dissimilar to Spero and Lea (1996) who noted that *G. bulloides* tended to grow their chambers over the first 4 to 5 days in culture. As the majority of culturing studies on *G. bulloides* end by day 10 (e.g. Mashiotta et al., 1997; Lea et al., 1999; Hönisch et al., 2011) it is not possible to determine whether the reported short growth period is a feature of all *G. bulloides* or whether it is a function of their short lifespan in previous cultures. The results of this study do however support the conclusion that it may be the former. The comparable size increase between this study and former studies feeding *Artemia* spp. nauplii implies that diet has had limited impact on growth and supports the decision to use microalgae for easier management of the specimens.

The significant anticorrelation ($r$ = −0.546) between longevity in culture and the final axis length on death may reflect differences in resource management by the individuals. In response to being placed in culture it appears that the specimens were split between those that allocated energy to chamber addition and growth, versus those that utilised their resources for cytoplasm and spine maintenance, and a longer lifespan. Contrary to earlier publications, no individual was directly observed carrying out gametogenesis. There was however a distinct split between individuals that died through complete spine and cytoplasm loss (63%), and those which deteriorated slowly while retaining sparse and/or shortened spines even when no longer maintaining cytoplasm or rhizopods (37%). Previous studies characterised gametogenesis at death by the total loss of spines, positing that some degree of spine retention indicated its absence (Spero and Lea, 1996; Kuroyanagi et al., 2013, 2019). In line with the findings of Kuroyanagi et al. (2013), who reported a ratio of at least 60:40 for complete to non-complete spine loss at death, our study observed a comparable ratio of 63:37. It is therefore likely that early death was linked to reaching sexual maturity. If this is the case, then from the foraminifera’s perspective high mortality is not necessarily negative in terms of overall species survival.

It is unclear if there is a strategic benefit to the foraminifera for a prolonged lifespan with lower growth. It may be that by maintaining a state of inactivity, the individual aims to wait out less favorable conditions and that should conditions improve, growth or reproduction would begin (Ross and Hallock, 2016; Westgård et al., 2023). Work on benthic foraminiferal species found that greater stress lead to progressively longer lifespans, indicating a link between the two (Hayward et al., 2014). A study on the foraminifera *Ammonia tepida* (Cushman) found that growth and reproduction ceased outside of a preferred temperature range but restarted again upon transfer into “ideal” conditions (Bradshaw, 1957). That longer living specimens apparently did not grow beyond the initial culturing period, or show signs of reproduction at death, could imply that an environment these foraminifera considered “favourable” was never achieved. It is unclear what was unfavourable about the culturing treatments as the same phenomenon was observed under ambient (albeit stable) conditions. Individuals were recorded capturing food particles throughout the experiments (see *Feeding*), which alongside the elevated longevity indicates ongoing metabolic and biological activity. This was important for confirming that they were still living as foraminifera that die without reproduction are likely to withhold cytoplasm within the shell, making it harder to identify the actual point of death (Murray and Bowser, 2000). The prolonged decline of smaller, older specimens, compared to the swift growth and subsequent death seen in larger ones, therefore suggests that the latter invested all their energy into calcification, leading to rapid demise once resources are depleted, while the former could have succumbed to the gradual effects of age-related degeneration.

Specimens in pH 7.8 and CI77 treatments, despite having high initial mortality and considerably lower spine recovery and maintenance, had some of the highest median (323, 345 μm) and mean (318, 342 μm) longest axis lengths upon death. This suggests that their response to culture was to utilise resources for growth, and potentially reproduction, rather than to achieve longevity. Large final sizes and a short lifespan has previously been linked to low stress in benthic agglutinated foraminifera, with successively smaller sizes and greater lifespans in increasingly stressful environments (Hayward et al., 2014). Due to the low recovery observed in terms of spine regrowth or maintenance, combined with low rhizopodial activity, it is concluded that specimens of pH 7.8 and CI77 were not exhibiting this phenomenon, however, and that instead, their high mortality is attributed as being a stress response.

### Tolerance and adaptability to extreme conditions

*G. bulloides* are generally considered a subpolar to subtropical species, with abundance peaks in water temperatures of around 10–15 and 20°C (Kucera, 2007), and a range that covers salinities of around 34 to 37, with slightly lower values in the Pacific than the Atlantic Ocean (Kao et al., 2018). There are also clear differences in carbonate chemistry in the Pacific and Atlantic Ocean, with lower pH, [CO_3_^2−^] and calcite saturation in the Pacific water relative to the Atlantic (Takahashi et al., 2014; Jiang et al., 2015). A review of growth rate dependence on temperature suggests growth in *G. bulloides* should be severely inhibited below 10°C (Lombard et al., 2009). This set of culturing experiments shows however that they are highly resilient to conditions outside of their optimum habitat, with no significant difference in mortality rates or final longest axis in the low salinity, pH, or temperature treatments, relative to treatments closer to ambient conditions. Spine regrowth and maintenance after picking tells a similar story, however curiously the greatest tolerance and recovery was in two of the presumably most extreme treatments, pH 7.7 ([CO_3_^2−^] 71.0 μmol/kg) and S30.4, with 84% and 100%, respectively. In an earlier study spine recovery was directly correlated with pH, with specimens in the lower pHs having lower recovery rates than specimens in high pH water (Davis et al., 2017). This was likely related to their other finding that pH and [CO_3_^2−^] were correlated with the amount of calcification. No such correlation appeared to be present in this study (with no relation between [CO_3_^2−^] and final size), but when pH 7.8 and CI225 are excluded, our values reflect Davis et al.’s (2017) findings that above pH 7.7, spine recovery and maintenance was around 65 to 100%.

These results imply that *G. bulloides* can tolerate cooler, fresher waters beyond their thermal optimum (Kucera, 2007). While they can be found upon occasion in polar waters this is the result of the encroachment of Atlantic waters, as opposed to their being endemic to the region (e.g. Anglada-Ortiz et al., 2021). In the North Atlantic *G. bulloides* abundance is strongly linked to high productivity (Schiebel et al., 2001) suggesting that its expansion to high latitudes may not be completely limited by hydrographic properties but also food availability. Maintaining an extended array of spines and rhizopods has an associated energy trade-off (Gaskell and Hull, 2019) and therefore may be a negative selective pressure in the high latitudes where there is a highly variable and seasonal food supply (Caroll and Caroll, 2003). Assuming that spines are utilised as a buoyancy aid (Jon Furbish and Arnold, 1997), they may provide less benefit in cooler, denser waters, where buoyancy issues are less of a consideration for foraminifera (Rajeshwara Rao and Hussain, 2018). In combination this may act as a further inhibiter on the expansion of *G. bulloides* as their partitioning of resources for spine maintenance will put them at a competitive disadvantage without providing the same degree of associated benefits. This could change into the future as predicted increases in phytoplankton productivity (Arrigo and van Dijken, 2015), in combination with rising sea temperatures (Asbjørnsen et al., 2020), may facilitate expansion of *G. bulloides* northwards.

### Ectoplasmic structures

In the later stages of their lifecycle, some *G. bulloides* individuals began to use their spine and cytoplasm as skeletons for the construction of solid organic structures, comprising both foraminifera cytoplasm and biological matter scavenged from the culture medium. While observed in deep-sea benthic species (Wollenburg et al., 2021) this had not been reported in planktic species before Greco et al. (2023). Greco et al. (2023) found that ectoplasmic structures in the studied planktic foraminifera appeared as two different types of projection: either root- or twig-like, or filipodia-like. Only the twig-like structure was reported in *G. bulloides*, appearing on day 18 in culture and was interpreted as potentially having a role in increasing prey encounters for feeding. Our observations corroborate this initial report, with twig-like structures appearing in several specimens. These structures did not begin to form until at least day 16 and did not become fully developed until day 30 at the earliest, which given the generally short culturing period for *G. bulloides* in earlier work, would likely explain why they have gone unreported before. It was theorized in the case of deep sea benthic species that ectoplasmic twigs provide a supportive framework from which the rhizopod network can extend and catch food (Wollenburg et al., 2021). As the aggregated particles within the structures on *G. bulloides* were retained and not consumed it supports the theory that they themselves were not the food source and may have had a role stabilizing and reinforcing the spine and rhizopod network. Having spines already decreases sinking speeds relative to non-spinose species (Takahashi and Be, 1984) and so by further increasing the specimen’s surface area, and thus fluid drag, the ectoplasmic twigs may help in maintaining position for feeding or reproduction. This would be particularly important for older, larger specimens that have a negative buoyancy (Meilland et al., 2021). Long spines, while more effective at producing drag, are also more susceptible to mechanical breakage (Jon Furbish and Arnold, 1997), so the stabilizing effect of aggregated particles along their length may also be working to protect the spines from damage. This may be of particular importance in the latter stages of a foraminifera’s life cycle when the individual is no longer able to expend the same biochemical energy required to maintain other forms of buoyancy such as low-density lipids or gases (Jon Furbish and Arnold, 1997).

### Implications for palaeoceanography

It can be understood from these results that larger specimens live for a shorter time period. From a palaeo reconstructions perspective this is critical as it would mean that larger size fractions in the fossil assemblage record a different climate signal to the smaller size fraction. Where short lived specimens may be reflective of the environment over the space of a few weeks, long lived specimens could carry the climate signal of an entire season. Calcite in large shells of *G. bulloides* has been repeatedly observed as having higher δ^18^O values than in smaller size fractions (e.g. Peeters et al., 2002; Jonkers et al., 2013). This has been put down to ontogenetic effects, or differences in average calcification depth (Peeters et al., 2002), but may also signify growth in different time-integrated temperature and seawater δ^18^O signals. ^18^O values in foraminiferal calcite decreases from winter to summer in tandem with rising sea temperatures (Austin et al., 2006; Wan et al., 2010). Assuming a foraminiferal bloom in Spring (Jonkers et al., 2013), it can be interpreted that larger, shorter living specimens of *G. bulloides* are growing and dying in colder water than the smaller specimens, which will continuing living and calcifying through the summer months. The difference in seawater temperature may be thus reflected in the final δ^18^O values of the shell calcite in the different size fractions.

The retention of spines in mortality via slow decay versus complete spine loss may have consequences for Na/Ca-proxy calibrations. Na incorporation in shell calcite is positively related to salinity (Mezger et al., 2018) with particular enrichment in the spines (Mezger et al., 2019). Fossil sediment samples of spinose foraminifera are usually lacking the fragile spine component however due to breakage. It was found that in bulk specimen analysis of *Globigerinoides ruber* and *Trilobatus sacculifer*, calcite Na/Ca decreased with water depth until reaching levels that corresponded with core top samples. This was hypothesised as resulting from the loss of high Na/Ca spines as the foraminifera sinks through the water column upon completing its life cycle (Mezger et al., 2018). As 37% of our samples retained spines to some degree this means that any Na based culture calibration performed on these specimens would likely display elevated Na/Ca relative to fossil specimens from corresponding water conditions. Assuming this is a phenomenon common to culturing (e.g. Kuroyanagi et al., 2013 also observed that up to 40% of specimens died without dropping spines), it underscores the need for caution when applying culture-based Na/Ca calibrations to fossil *G. bulloides* for salinity reconstructions, especially if the calibration is based on bulk solution analyses.

Furthermore, the development of spines into ectoplasmic twigs may have consequences for shell based geochemical reconstructions by altering the microenvironment for calcification or increasing the dissolution potential requiring more energy for calcite growth. While spinose species tend to incorporate Ba in a ratio reflecting seawater [Ba], non-spinose species have been found to be enriched with Ba relative to the ambient concentration (Lea and Boyle, 1991; Richey et al., 2022). In *Neogloboquadrina dutertrei* and *Globorotalia truncatulinoides*, calcification within an organic aggregate microenvironment was theorised to be the cause of elevated Ba/Ca ratios due to Ba release from biogenic particles (Fehrenbacher et al., 2018; Richey et al., 2022). In ocean dwelling *G. bulloides*, fossils from longer living specimens that developed ectoplasmic structures may also display elevated Ba/Ca ratios relative to non-ectoplasmic specimens. As further micro-environmental parameters, such as pH and [CO_3_^2−^] were found to play no role in Ba partitioning in *G. bulloides* (Hönisch et al., 2011), variation in Ba/Ca in fossil specimens from an environment where seawater Ba is expected to have stayed constant, and seafloor Ba incorporation negligible, can potentially indicate the presence of ectoplasmic structure. It first needs to be confirmed, however, whether ectoplasmic specimens of *G. bulloides* have an elevated Ba/Ca ratio relative to non-ectoplasmic specimens grown in the same treatment water.

## CONCLUSION

We performed an extensive set of culturing experiments on the temperate to subpolar planktic foraminiferal species *G. bulloides*, including low temperature (6–13°C) and variable salinity and carbonate chemistry experiments. *G. bulloides* were observed to survive up to three months in culture, potentially as a function of a microalgal diet instead of the more widely used *Artemia* spp. nauplii. As feeding microalgae supports cultivation of much larger sample sizes, important to robust statistical results and conclusions, we recommend the use of microalgae in future studies.

*G. bulloides* was determined to be tolerant of environmental conditions that were well outside its natural optimum range (<10°C, salinity <33, pH <8), with no significant difference in test size on death, between any of the different treatments. This suggests a high degree of adaptability that may serve it well under future ocean warming and acidifying scenarios in the Nordic Seas (Fransner et al., 2022; Saes et al., 2022). Its resilience in the low-salinity treatment suggests it will also likely tolerate local freshening events due to changes in ocean circulation and freshwater fluxes (Glessmer et al., 2014; Mork et al., 2019).

We observed two apparent strategies employed by individuals in response to culture conditions. The first was a rapid increase in size followed by complete spine loss and death. The second was prolonged survival, with continued biological activity but minimal growth, followed by cytoplasm and spine decay to the end of life. The first is likely the result of death via sexual reproduction while the latter would represent death without a reproductive event. Although gametogenesis was never directly observed this is possibly an artifact of observation frequency and should not be taken to mean it did not occur. From a palaeo perspective the two different life strategies mean that the large shell size fraction in a sediment sample might represent calcification over a significantly shorter timespan than the smaller size fraction, which could have a signal integrated over several months. This may explain why larger shells of *G. bulloides* are reported as having higher δ^18^O values than smaller size fractions (Jonkers et al., 2013). Furthermore, the retention of spines in longer living specimens may lead to a positive bias in Na/Ca values in cultured specimens where spines are not completely lost before analysis, leading to erroneous application in reconstruction studies. The impacts of the biological behaviors observed here on shell geochemistry therefore warrants further investigation as they are likely to have significant implications for palaeoceanographic reconstructions using *G. bulloides*.

## Data Availability

Data are available on request to the main author until publication on theCristin database (https://www.cristin.no/).
